# Nanohardness and Residual Stress in TiN Coatings

**DOI:** 10.3390/ma4050929

**Published:** 2011-05-17

**Authors:** Luis Carlos Hernández, Luis Ponce, Abel Fundora, Enrique López, Eduardo Pérez

**Affiliations:** 1 Laboratory of Laser Technology, CICATA-IPN, Altamira Unit, México C.P 89600, México; E-Mail: guira_98@yahoo.com; 2 Institute of Science and Technology of Materials (IMRE), Havana University, La Habana, Cuba C.P 10400, México; E-Mail: fundora@fisica.uh.cu; 3 Centro de Innovación, Investigación y Desarrollo en Ingeniería y Tecnología (CIIDIT), Universidad Autónoma de Nuevo León, Monterrey, Nuevo León, México C.P. 66450, México; E-Mails: enlopez_73@yahoo.com (E.L.); eduardo.pereztj@uanl.edu.mx (E.P.)

**Keywords:** negative substrate bias potential, residual stress, nanohardness, nanoindentation

## Abstract

TiN films were prepared by the Cathodic arc evaporation deposition method under different negative substrate bias. AFM image analyses show that the growth mode of biased coatings changes from 3D island to lateral when the negative bias potential is increased. Nanohardness of the thin films was measured by nanoindentation, and residual stress was determined using Grazing incidence X ray diffraction. The maximum value of residual stress is reached at −100 V substrate bias coinciding with the biggest values of adhesion and nanohardness. Nanoindentation measurement proves that the force-depth curve shifts due to residual stress. The experimental results demonstrate that nanohardness is seriously affected by the residual stress.

## 1. Introduction

Titanium Nitride has been used as a hard wear protective coating, which has an excellent combination of good performance at room temperature and attractive appearance. TiN coatings production using Cathodic arc evaporation (CAE) deposition is an established industrial technology [1]. In the cathodic arc evaporation process, an arc discharge is initiated on a cathode surface (target material) inside a vacuum chamber. The high current density at the arc spot forms metal plasma, which contains a high density of metal ions as well as electrons, neutral atoms and spherical agglomerates called ‘macroparticles’ [2]. TiN films show different mechanical properties depending upon the deposition conditions: the substrate bias voltage [3], the substrate temperature [4],and the nitrogen pressure [5]. In general, a negative bias voltage (*V*B) is applied to the substrate during deposition [6] to enhance the hardness (H) and adhesion of the TiN coatings, attributable to the ion bombardment energy [7].

Classical indentation hardness measurements are now widely used for the mechanical properties characterization of hard coatings because they are simple, economical and reproducible [8]. Since the thickness of these hard coatings starts at a few micrometers, an accurate measurement of the mechanical properties by the microindentation technique is not easy, because the results can be affected by the substrate. Due to a gradually increasing influence of the substrate, in this case AISI 410 stainless steel, the hardness properties of composite systems depends possibly not only on TiN coating, but also on coating thickness and substrate hardness. The control of the indentation depth is the main problem of classical indentation, because depths less than 1–2 μm make the indentation too small for measurement by conventional microscopes [9]. In the case of thin films, their intrinsic hardness becomes relevant only if the influence of the substrate material can be eliminated. Thus, the indentation depth should not exceed about one-tenth of the total coating thickness.

The mechanical properties of TiN coatings with thicknesses of the order of micrometers have been widely reported in the literature [10]. In the present work we will study the TiN hardness properties close to the coatings surface with accuracy in the range of nanometers by a nanoindentation tester. Usually, the principal objective of such testing is to obtain the films hardness values from experimental readings of indenter load and depth penetration. Nanoindentation is simply an indentation test in which the length scale of the penetration is measured in nanometers rather than microns or millimeters, the latter being common in conventional hardness testing. In a nanoindentation test the forces involved are in the range of milli-Newtons (mN) to a few nano-Newtons.

The goal of this work is to study, surface morphology, residual stress, adhesion and nanohardness as a function of negative substrate bias. For this reason, the coating thicknesses are measured by the Calotest technique. Atomic force microscopy (AFM) is used to observe the surfaces of as-deposited TiN coatings and to correlate the effects of ion current on enhancement of the surface morphology. The residual stress in the different TiN coatings is measured by the Grazing incidence X-ray diffraction technique. The Scratch test is reported in order to study the adhesion properties of TiN coatings on AISI 410 stainless steel substrates.

## 2. Experimental procedure

### 2.1. The Coatings Preparation

To enhance the mechanical properties of TiN coatings in the present work, the ionized particles are accelerated towards the AISI 410 stainless steel substrate applying a negative bias voltage (0, −100 V, −300 V). A cathode arc source of high purity Ti (99.9%) and ultra-high purity nitrogen and argon gases were used. The voltage and current of each cathode arc source was 20 V and 70 A, respectively. The temperature was kept constant at 400 °C during depositions. Prior to the deposition process, AISI 410 substrates were polished using different sandpapers from 220 to 600 grit. The substrates were also cleaned in successive ultrasonic baths of acetone and then isopropyl alcohol.

### 2.2. Surface Morphology Observations

AFM was used to observe the surface morphology of different TiN coatings. Manipulation experiments were carried out using a Veeco Instrument Multimode scanning probe microscope in hard tapping mode (low amplitude set point voltage). This method minimized surface contact and lateral forces by oscillating the cantilever at 285 kHz (≈1 nm root mean square), resulting in a significant improvement in lateral resolution on soft surfaces. The AFM images were recorded by scanning the sample at room temperature (25 °C), inside of 1 μm^2^ with a scan rate of 1Hz. Image Processing and Data Analysis software (Version 2.1.15) by TM Microscope was used for image analysis.

### 2.3. Mechanical and Tribological Characterization Methods

#### 2.3.1. Calotest

**Figure 1:**
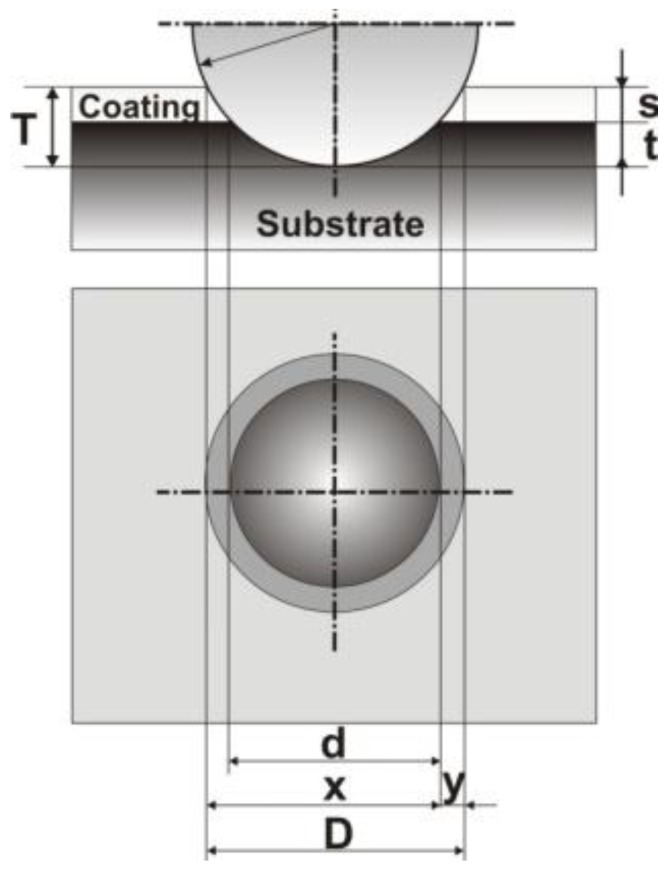
Calotest measurement scheme.

The Calotest is an established method to determine the coating thickness. In this work, Calotest equipment of CSM Instruments was used. In general, a stainless steel ball (~0.02 m diameter) is used with diamond paste to wear through the coatings surface until the substrate has been reached by the ball. The coating is placed under an optical microscope where the circular crater shape is used to measure the coating thickness by comparing the relative diameter of the exposed surface layers to the known diameter of the ball. Figure 1 shows the Calotest measurement scheme where **T** is the total penetration depth, **t** is the substrate penetration depth and **s** is the thickness of the coating.

By measuring the parameters ***x*** and ***y***, the thickness of the coating can be calculated by a simple geometrical equation [11].

(1) $$s=\frac{xy}{2R}$$

#### 2.3.2. Determination of Residual Stress by X-Ray Diffraction

TiN diffraction patterns for all coatings were recorded. X-ray patterns showed polycrystalline films with diffraction peaks for the crystalline planes: (111), (200), (220) and (222). The coatings displayed mainly a (111) preferred orientation. For residual stress measurements it is recommended to use high diffraction angles, 2θ > 100°, and high multiplicity peaks (hkl) in order to maximize the peak shift due to stresses, to lower the effects due to misalignment and to sample a larger number of lattice planes

The TiN coatings residual stresses were measured using a Rigaku Strainflex diffractometer (Cr–Kα radiation) in parallel beam geometry [12]. The sin^2^ψ method was used to evaluate the data. The analysis was performed at high diffraction angles, 130° < 2θ < 150°, on the TiN (222) reflection with two ψ tilts 0° and 70°.

The residual stress (***σ***) was obtained from: ***σ*** *= **m K_1_*** (2), where ***m*** is the slope of 2θ *vs*. sin^2^ψ curve and stress constant ***K_1_*** is −1,700 MPa/°.

#### 2.3.3. Scratch Test

The scratch testing method is used to evaluate the adhesive behavior (scratch toughness) of the coatings. Tests were carried out in the CSEM Revetest device. The diamond stylus, with a tip radius of 0.2 mm, was loaded against the coated substrate with a loading rate of 100 N/min and a horizontal displacement rate of 10 mm/min. The lower critical load (L_c_) is defined as the smallest load at which appreciable damage starts or the friction coefficient rapidly rises. By optical microscopy, the L_c_ value was determined.

#### 2.3.4. Nanoindentation Testing

The nanoindentation measurements were performed with an instrument consisting of a nanohardness tester (CSEM Instruments) and an integrated optical microscope. Both components are directly linked by an automated positioning system, which allows movements in the *X*-*Y* axes. Prior to the indentation, the sample is kept under the optical microscope and an appropriate area is chosen. Indentations were made on the desired area using a Berkovich diamond indenter. Standard and multi-cycling progressive load and in depth control nanoindentation methods have been used to evaluate the TiN coatings nanohardness properties. The hardness was calculated using the Oliver-Pharr analysis method [13], which could be executed from the software provided by CSEM Instruments.

Standard and multi-cycling progressive nanoindentations have been used to evaluate the TiN coatings nanohardness properties. For standard nanoindentation measurement, a diamond stylus (indenter) is pressed in the range of nanometers into a surface with a known load. The depth penetration under the films surface is recorded as the load is applied in the indenter with a prescribed loading and unloading curve. The maximum depth of penetration for a particular load, together with the slope of the unloading curve measured at the tangent to the data point at the maximum load, leads to a measure of material hardness.

**Figure 2:**
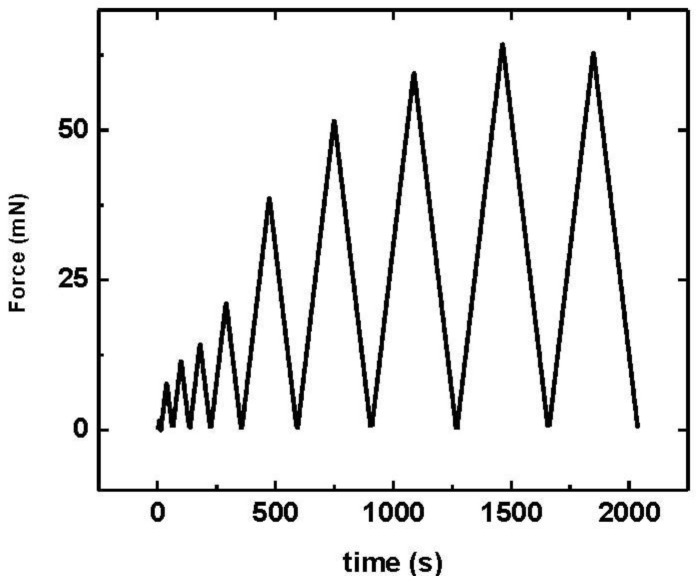
Profile of load/unload in multi-cycle progressive nanoindentations.

The multi-cycle progressive nanoindentations in depth control mode are used to measure the hardness distribution along the TiN thickness coating. In this measurement the depth of maximum penetration changes with the number of cycles. It is computed with the number of the cycles and the difference between the first and last max depth. In each cycle the loading rate is increased linearly until the maximum penetration depth is reached. After a stop of 2 s, the unloading rate begins to decrease linearly until finishing at zero load. The multi-cycle progressive nanoindentions were performed to 10 cycles. The first load cycle reached a max depth of 40 nm, and 800 nm in the last cycle. The loading/unloading rate was increased linearly at 20 mN/min, and the pause between cycles was 0.1 s. (as can be seen in Figure 2).

## 3. Results and Discussion

### 3.1. Thickness

**Figure 3:**
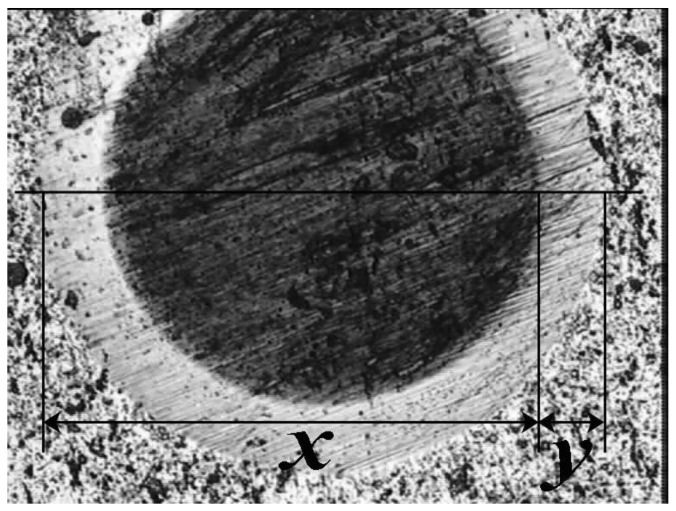
Ball crater produced in the TiN coating over AISI 410 stainless steel substrate.

Figure 3 shows the ball crater in the TiN coating and AISI 410 stainless steel substrate produced by the Calotest technique. The circular crater diameters are determined by the optical microscope and the coating thickness can be calculated according to Equation 1. The TiN coatings thickness values are reported in Table 1.

**Table 1:** The TiN thickness, residual stress and critical loads values of TiN coatings as a function of negative substrate bias potential.

Bias substrate (V)	Thickness ( μm )	Residual stress (GPa)	L_c_ (N)
0	3.3	−0.3	6.6
−100	3.2	−11.0	16.3
−300	2.6	−3.0	14.4

As shown in Table 1, the TiN thickness films decreased when the bias applied to the substrate was increased. This result can be explained as a consequence of the higher bias polarization providing energy to the system which enhances the surface mobility of all particles on the layer surface. Hence, atoms in the film occupy new compact lattice sites which could not be reached with lower impact energy.

### 3.2. AFM Image Analysis

**Figure 4:**
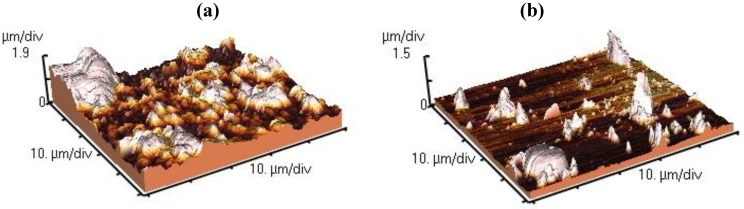
Afm images and *R*_rms_ of TiN Coatings: **(a)** 0 V and **(b)** −300 V.

TiN coatings surface topography at 0 V and −300 V are shown in the Figure 4. The AFM images show clear differences between surface topography, with the presence of island growth at 0 V, whereas at −300 V a uniform and smooth surface forms (typical of lateral growth mode).

**Figure 5:**
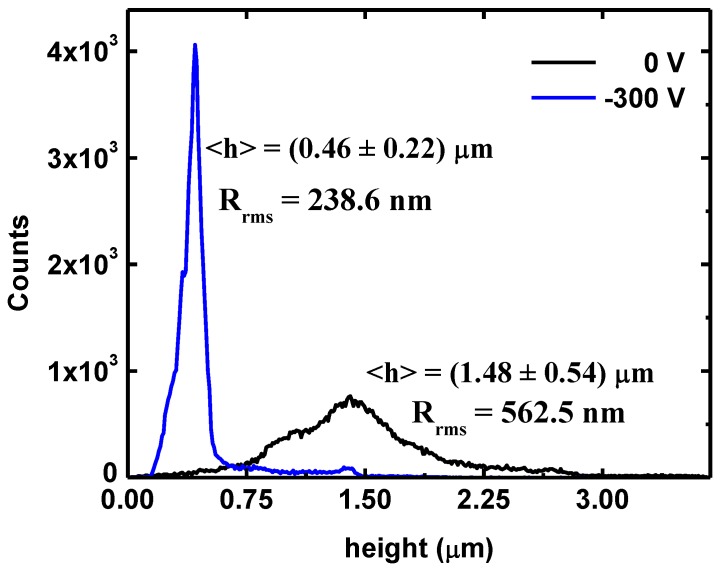
The height distribution at different bias voltages: 0 V, 100 and −300 V.

The AFM height data were statistically analyzed by the distribution, mean and standard deviation. The root mean roughness of the surface (*R*_rms_) was also studied [14]. Figure 5 shows that the height distribution is more narrowly scattered for −300 V than 0 V. Additionally, it can be observed that the mean height and the *R*_rms_ diminish with the increase in the negative bias potential.

From qualitative and quantitative analysis of TiN coatings AFM images, we can summarize that the growth mode changed from a 3D island growth to a lateral growth when the bias potential was increased. The explanation of this phenomenon is supported by bombardment effects of the growing films by energetic particles, which enhance the surface mobility of the condensed species, promoting the displacement of surface atoms towards more stable positions in terms of surface energy, and resulting in the elimination of voids, cavities, and vacancies in the coatings [15].

### 3.3. Residual Stress and Adherence

The residual stress and critical load as a function of the substrate bias potential, in the range from 0 to −300 V, is summarized in Table 1. As it is shown the compressive stress rises with increasing bias from −0.28 GPa to about −11 GPa, for the coatings deposited at 0 V and −100 V, respectively. A similar increase in stress with bias voltage has been already reported for the TiN coatings [12,16,17]. The effect of bias polarization enhances the surface mobility of all particles due to either the increase of the atoms kinetic energy, or the collisions between the energetic particles that reach the layer surface. With this higher mobility of the atoms, voids collapse to dimensions comparable to the range of interatomic forces and the stresses tend to compressive [18]. Moreover, on increasing bias voltage from −100 V to −300 V, a reduction in compressive stress to 2.97 GPa is observed. The decrease in the compressive stress could be due to several effects: the formation of the layered structure, the thermal relaxation of the structure and, the enhancement of the atom mobility [19].

**Figure 6:**
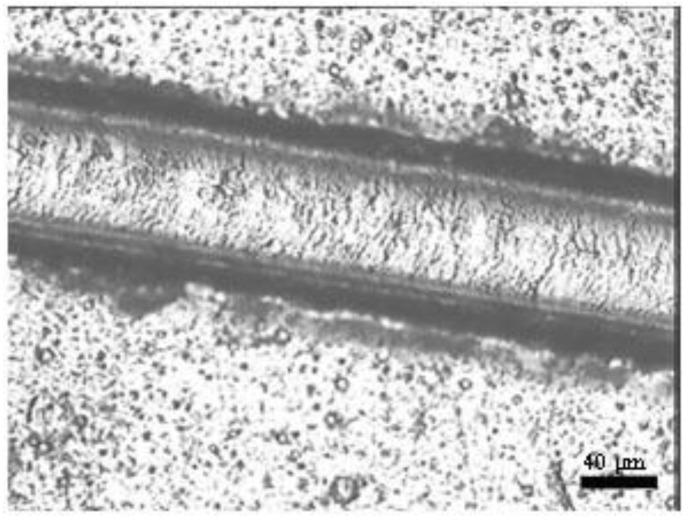
Micrographs of failure events used in scratch testing for the TiN coatings with −300 V bias potential.

An adhesive failure micrograph for the coating with −300 V bias potential is shown in Figure 6. In accordance with the literature report [20], in all samples the failure mechanisms started from the several spallings on both edges of the developed scratch. The difference was in the position of these spallings. In the case of the −300 V, spallings began from a load above 14 N. As the load increased, some large lateral cracks started to appear, which became more frequent at higher loads. This failure mechanism in the TiN coating is the result of complete delamination (adhesive failure) due to the compressive stress field produced by the moving stylus. Partial spallation occurred earlier in the track and continued until total failure occurred without any significant changes.

The adhesion strength between film and substrate is strongly affected by the residual stresses in the film as it is reported in reference [21]. As shown in Table 1, the critical load exhibits analogous behavior to the residual stress. The critical load increases from 6.6 to 16.3 N when the bias substrate rises from 0 to −100 V and decreases to 14.4 N for −300 V. Therefore, the adhesion at negative bias substrate is influenced by the compressive stresses produced during deposition.

### 3.4. Nanohardness Properties of TiN Coatings.

**Figure 7:**
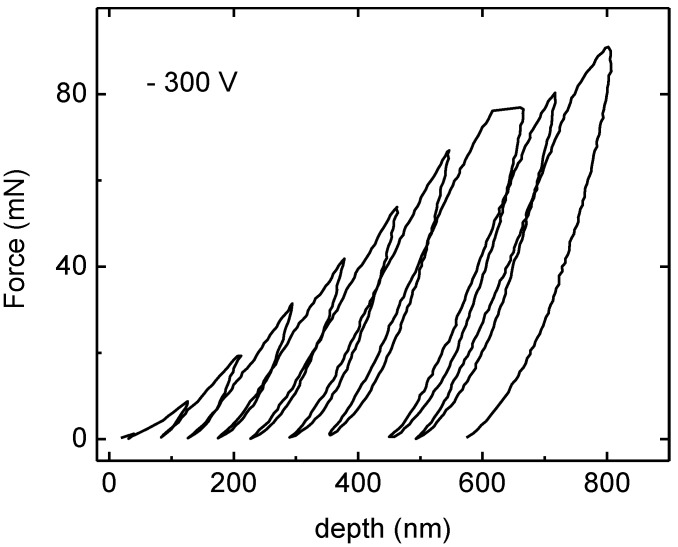
TiN Force-depth curve for multi-cycle progressive load in depth mode for coatings with −300 V bias substrate.

Figure 7 displays the typical multicycle progressive load–depth curve in depth mode for TiN coatings with −300 V. Multicycling testing refers to performing a sequence of ten loading–unloading cycles in one experiment, thus saving time and providing improved repeatability. In each cycle, the unloading slope from the force-depth curve is analyzed by the Oliver and Pharr methods to determine the coatings hardness. In Figure 7, a small viscoelastic rebound can be observed, which causes hysteresis loops to appear in the unloading-reloading data.

**Figure 8:**
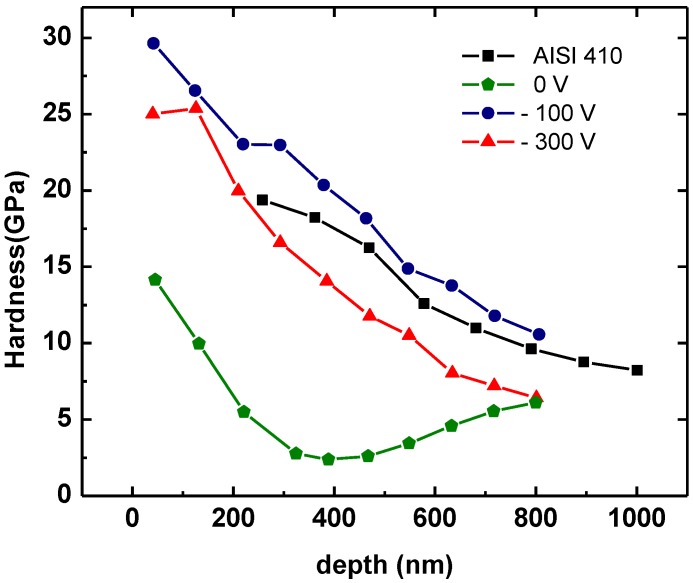
TiN coatings and AISI 410 substrate hardness values of each multi-cycle load plotted as a function of penetration depth.

The hardness values of each multi-cycle load plotted as a function of penetration depth for both TiN coatings and the steel substrate are displayed in Figure 8. This figure reveals that the hardness of TiN coatings decreases with the penetration depth. This nanohardness-depth profiling can be explained by the residual stress effect. In the literature it has been previously reported that TiN hardness is enhanced when the compressive stress is increased [22]. Also, residual stress has been recently measured in situ during the TiN deposition process [23], and a decrease of compressive stress with further film growth was observed. The explanation is based on the origin of stress gradients arising from two competing stress generation mechanisms, namely growth-induced point defects (compressive stress) as a consequence of atomic peening, and void formation (tensile stress) as a consequence of surface roughness and shadowing effects. Both literature reports conclude that the nanohardness-depth profiling is strongly related to the residual stress generated during the coating growth.

It is generally accepted that the ratio of the coating thickness to the indention depth should be larger than ten [24], in order to obtain hardness values without the influence of the underlying substrate. The hardness of biased samples is always higher than the substrate until the penetration depth reaches a critical value of 300 nm, which is around 1/10th of the coating thickness. Therefore, we assumed 300 nm as critical value of indentation depth for our samples.

**Figure 9:**
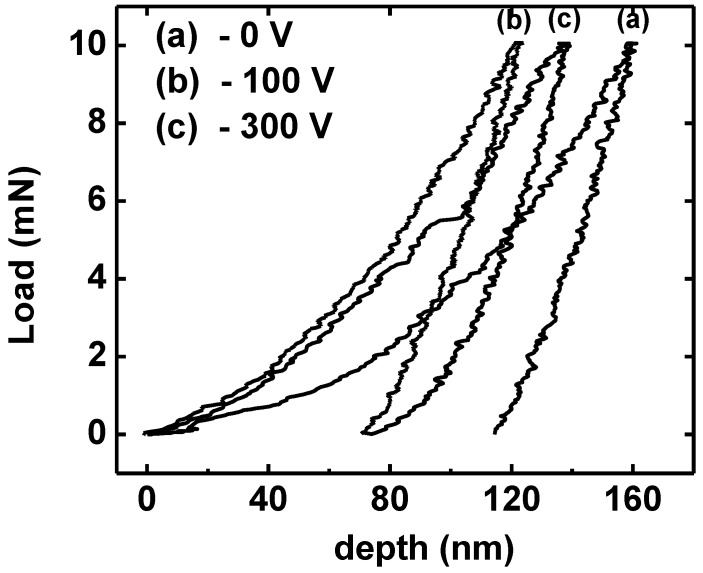
Force-depth curves of standard nanoindentation test for TiN coatings with different bias voltage. (**a**) 0 V; (**b**) −100 V and (**c**) −300 V.

The TiN coating at −100 V exhibits the higher hardness values and to explain this behavior, standard nanoindentation measurements were performed using a load of 10 mN to avoid substrate effect. The load-depth curves for the TiN samples are shown in Figure 9. The nanohardness was computed for each unloading curve (a)–(c) with values of 17.6, 31.0 and 23.3 GPa, respectively. As can be observed, the highest value of nanohardness corresponds to grown coating at −100 V bias substrate, the same film that shows maximum residual stress (see Table 1). Hence, we can conclude that nanohardness is seriously affected by the residual stress.

From load-depth curves it is possible to confirm the nature of residual stress in nanohardness measurements [25]. Figure 8 shows that the loading curves with residual stress deviate to the left from the film unstressed sample (without bias, see Table 1) meaning that compressive stress is present. The hardness enlarges with the increasing compressive stress that is believed to be due to film densification and to the presence of lattice defects in the film’s structure, which acts as an obstacle for dislocation motion.

## 4. Conclusions

Several TiN coatings on AISI 410 stainless steel substrates were produced by the arc-evaporation technique by applying to the substrate different negative bias voltages. Using the Calotest technique the coating thickness was determined; with the result that it diminishes when the bias applied to the substrate was increased. AFM image analyses showed that with an increase in the negative bias potential, the growth mode of biased coatings changed from 3D island to lateral, which led to a uniform and smooth surface morphology.

The residual stress was calculated for all samples by Grazing incidence X-ray diffraction, with the maximum value at −100 V. Using the scratch testing method, we also demonstrated that adhesive behavior of the TiN films on AISI 410 stainless steel substrates is influenced by the residual stress during deposition.

The hardness properties were investigated in a nanometric scale by the nanoindentation technique. The nanohardness measurements by multi-cycle progressive load, showed that hardness of TiN coatings tended towards the substrate hardness value when the penetration depth was increased. The results also indicated that in our coatings growth with negative bias voltage; the critical value of indentation depth should be below 300 nm. We conclude that nanohardness is seriously affected by the residual stress because the TiN coating at −100 V matches the higher hardness and compressive stress values.
